# The status of occult HBV infection in a high endemic region: risk of community HBV transmission and reactivation

**DOI:** 10.1186/s13104-025-07337-6

**Published:** 2025-07-01

**Authors:** Hussein Mukasa Kafeero, Ponsiano Ocama, Dorothy Ndagire, Abdul Walusansa, Mariam Namusoke, Ali Kudamba, Fahad Muwanda, Hakim Sendagire

**Affiliations:** 1 Department of Biomedical and Mechatronics Engineering, Kyambogo University, P.O Box 1, Kyambogo, Uganda; 2 Department of Medicine, College of Health Sciences, Makerere University, P.O Box 7062, Kampala, Uganda; 3 Department of Plant Sciences, Microbiology and Biotechnology, College of Natural Sciences, Makerere University, P.O Box 7062, Kampala, Uganda; 4 Department of Medical Microbiology, Habib Medical School, Faculty of Health Sciences, Islamic University in Uganda, P.O Box 7689, Kampala, Uganda; 5 Department of Biochemistry, Habib Medical School, Faculty of Health Sciences, Islamic University in Uganda, P.O Box 7689, Kampala, Uganda; 6 Department of Physiology, Habib Medical School, Faculty of Health Sciences, Islamic University in Uganda, P.O Box 7689, Kampala, Uganda; 7 Department of Microbiology, College of Health Sciences, Makerere University, P.O Box 7062, Kampala, Uganda

**Keywords:** Occult hepatitis B infection, Latency, Reactivation, Immunocompromised, Endemicity

## Abstract

**Objectives:**

Occult hepatitis B virus (OBI) infection, characterized by the presence of HBV DNA in the absence of detectable HBsAg in the blood, is considered a potential hidden pathway for HBV transmission and reactivation, which can lead to liver cancer. This study aimed to assess the prevalence of OBI in a region of Uganda with high HBV endemicity, in order to help explain variations in HBV distribution within the country.

**Results:**

Among the 387 participants who tested negative for HBsAg, the majority were women (240 individuals, 62.0%), married (242 individuals, 62.5%), and aged 30 years or older (207 individuals, 53.5%). The OBI was detected in 21 participants (5.43%). Most of those with OBI were 30 years old or younger (13 individuals, 61.9%), male (12 individuals, 57.1%), had normal liver enzyme levels, and showed an average viral load of 194.4 IU/mL with a standard deviation (SD) of ± 122.05.

**Supplementary Information:**

The online version contains supplementary material available at 10.1186/s13104-025-07337-6.

## Introduction

The latency of the hepatitis B virus (HBV) genome in the liver cells as covalently closed circular DNA (cccDNA) without the presence of the Hepatitis B surface antigen (HBsAg) in serum is called occult hepatitis B virus infection (OBI) [1]. Among the immune competent persons, OBI is under the immune control posing minimal threats [2]. However, among the immune compromised, reactivation of the virus may occur challenging the clinical management of the HBV [3].

There is limited data on the prevalence of the OBI in Uganda. However, it is influenced by the geographical location, infecting genotype(s), mutations in the pre-s/s gene, host immunity and co-infection with other virus(es) [4–6]. Globally, the prevalence of the OBI is estimated at 8% [7] with the World Health Organization (WHO) African region having the highest prevalence of 14.8% [8]. In Uganda, little is known about the prevalence of the OBI because of the stringent inclusion criteria [6].

The presence of the HBcAb (hepatitis B core antibody) alone or in combination with HBsAb (hepatitis B surface antibody) among the HBsAg (hepatitis B surface antigen) seronegative have been used as surrogate markers for the diagnosis of OBI in the absence of the nucleic acid based techniques (NATs) especially in resource constrained areas where HBV is endemic [9]. Using these serological biomarkers, OBI may be considered as seropositive OBI or seronegative OBI [10]. It is seropositive OBI when HBcAb alone is present or along with HBsAb and this accounts for 80–99% of all the OBI cases. In contrast, it is seronegative OBI when both HBcAb and HBsAb are negative. This accounts for only 1–20% of the OBI leavings NATs as the plausible alternative for its detection [10].

Due to cost implications in resource limited settings, the NATs cannot be used routinely leaving some of the OBI cases undiagnosed. This increases the risk of reactivation during immune suppression [11], the HBV transmission during blood transfusion [12], the mother-to-child transmission [13] and, or during transplantation of tissues and organs [14].

This study aimed at establishing the prevalence of the OBI among the HBsAg seronegative hospital attendees from a high HBV endemic region of Uganda to inform the way forward in the management of HBV in our country.

## Materials and methods

### Study site, design and population

**Fig. 1:**
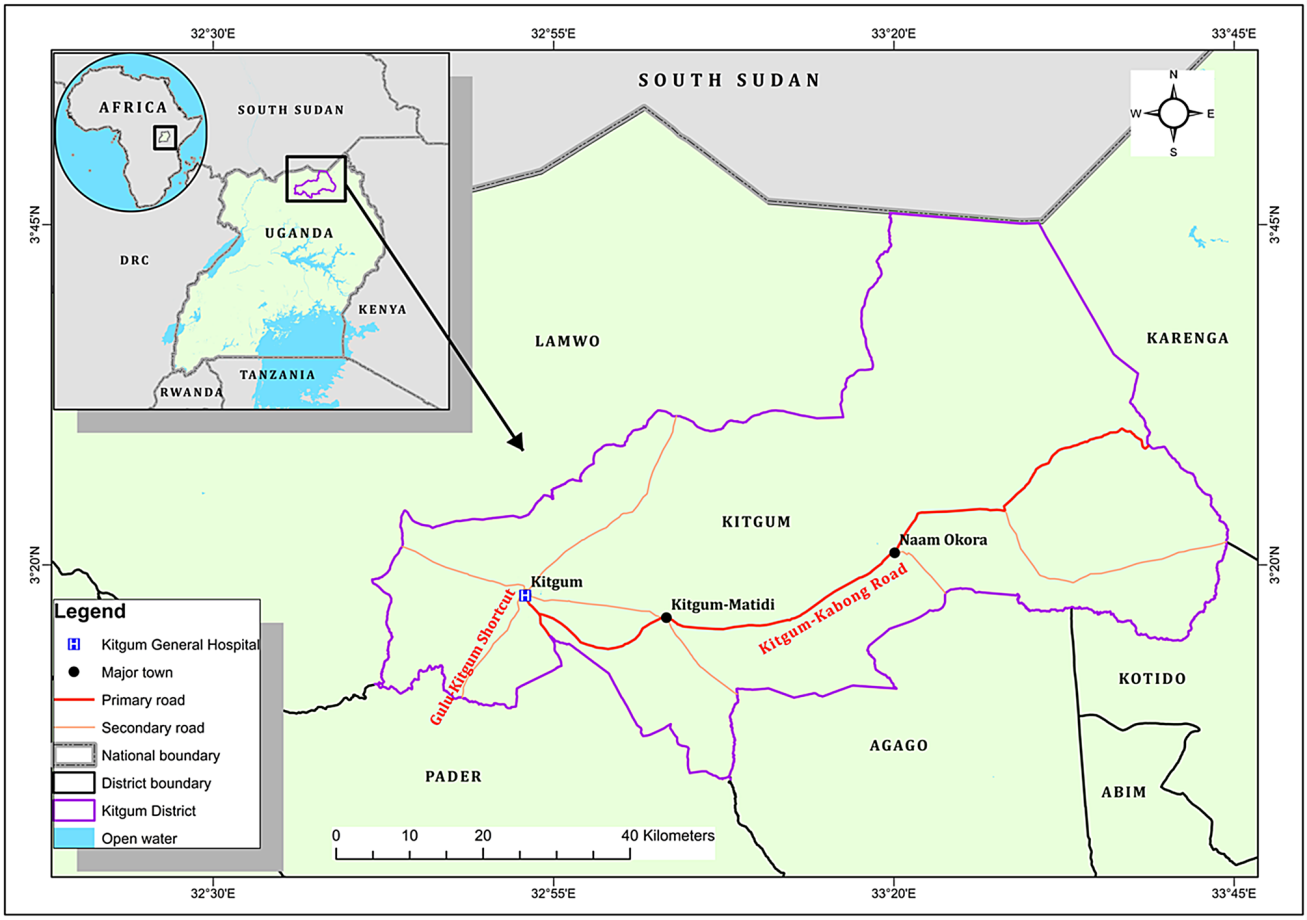
Study site

A hospital-based, cross-sectional study among adult outpatients (age ≥ 18) coming to Kigtum Hospital in Kitgum district and from the neighboring districts of Lamwo, Pader, Agago, Kotido and Kalenga was conducted between January to September 2020 at Kitgum General Hospital in Kitgum district, Northern Uganda (Fig. 1). Kitgum district is sparsely a populated with 204,012 people occupying an area of 3,960km^2^ with a population density of 62.6/km^2^ and an elevation of 760 m [15].

## Sample size determination and sampling procedure

The formula described by Cochran was used for the estimation of the sample size of 387 [16] at a prevalence of 50% and a standard normal deviation corresponding to the critical region of 1.96 at 5% precision. Consecutive purposive sampling was performed and any HBsAg seronegative hospital attendee after screening was eligible for inclusion in the study.

## Data collection tools and procedure

Information on the general characteristics and risk factors for the HBV infection of the participants was collected by using a close-ended pre-tested questionnaire. This was administered by a nurse or research assistant on site through a face-to-face interview. For laboratory investigations, 4mls of venous blood were drawn aseptically by vane puncture into disposable vacutainers in duplicates: - one for serum and another for whole blood. Both samples were stored at -4 °C in the hospital freezer pending cold chain transport to the study laboratories where they were stored at -20^o^C until further use.

### Screening for HBsAg serostatus and laboratory procedures

Screening for HBsAg status was conducted on-site using finger-prick blood samples and the HBsAg Rapid Test Strip (Healgen Scientific, Houston, USA) to determine participants’ eligibility for inclusion in the study. To qualitatively assess additional hepatitis B markers: HBsAb, HBeAg, HBeAb, and HBcAb, serum samples were tested using the 5-panel HBV One Step Combo Test Device (BIOZEK Medical, Apeldoorn, The Netherlands). The complete serological testing protocol is detailed in our previous publication [17].

Quantitative detection of the HBV DNA was carried out using the HBV assay by Roche Molecular Systems (Pleasanton, CA, USA) and Abbott Molecular (Des Plaines, IL, USA), which has a sensitivity threshold of ≤ 10 IU/mL. This assay identifies HBV DNA in serum regardless of genotype or sequence variation and is PCR-based, performed using the fully automated Cobas^®^ 4800 System. The procedure followed the protocol described by Chevaliez et al. [18]. Real-time PCR amplification and product detection were conducted using the Cobas TaqMan 96 analyzer, and data were processed using Amplilink software. The HBV DNA concentrations were reported in international units per milliliter (IU/mL).

Liver function tests were conducted using the B120 Chemistry Analyzer (Mindray, Nanshan, Shenzhen, China). Reference ranges provided by the manufacturer were applied: AST < 30 U/L, ALT ≤ 40 U/L, GGT ≤ 48 U/L, and serum bilirubin ≤ 17 µmol/L.

### Data quality assurance

Aseptic techniques and cold chain transport of the samples were ensured to maintain the potency of the sample. A one-day training session was given to the data collectors to ensure consistency during data collection. Finally, the screening for the HBsAg serostatus was performed twice: first at the hospital using the HBsAg Rapid Test Strip (Healgen Scientific Limited Liability Company, Houston, TX77047- USA). Second in the laboratory using the 5-panel HBV One Step Hepatitis B Virus Combo Test Device (BIOZEK, Medical; Apeldoorn, The Netherlands).

### Data processing and statistical analysis

The data collected were checked for completeness and consistency. After, the data were entered into the spread sheet in MS Excel and exported in SPSS version 26 or MedCalc 20.010 and cleaned for analysis. Frequency and summary statistics were used for categorical data. The differences in the demographic and Laboratory features of Patients with OBI and participants negative for the HBsAg and the HBV DNA were presented as either proportions or means with standard deviations. Comparison of proportions for categorical data was used for analysis. Numerical data was analyzed by using Mann Whitney U test due to lack of normality as tested by Chapiro Wilk test. Variables with *p* < 0.05 were considered to be predictors of occult hepatitis B virus infection.

## Results

**Fig. 2:**
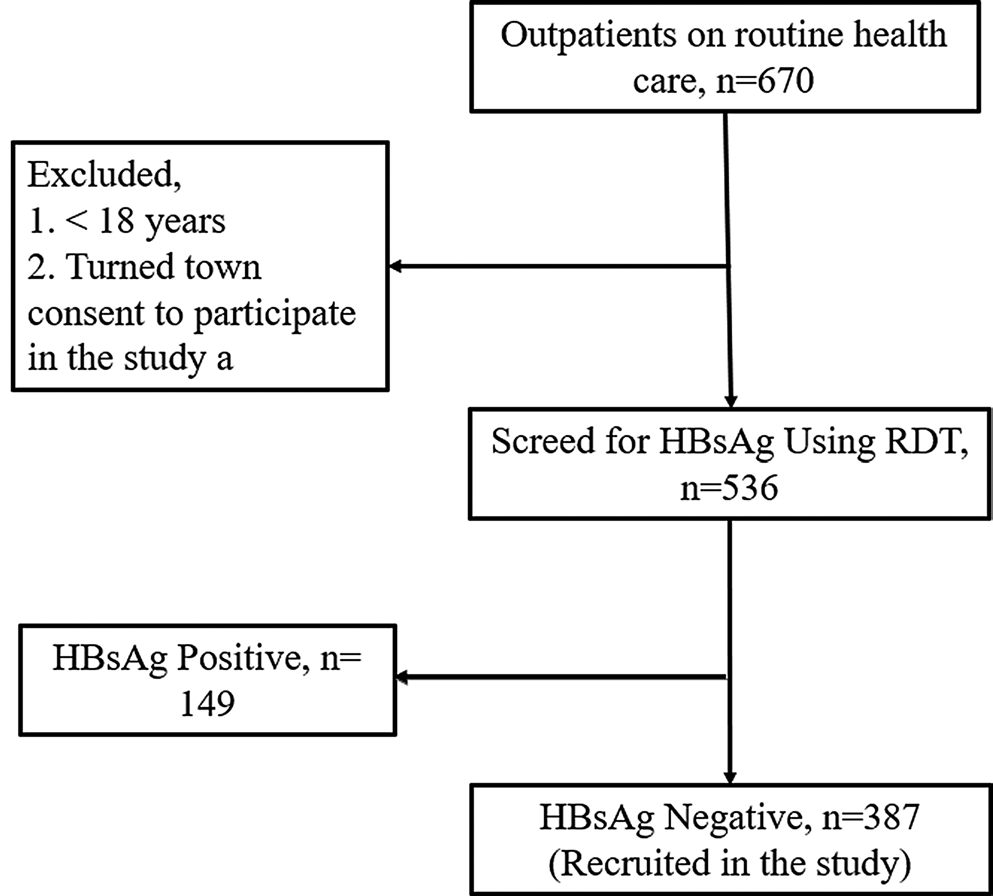
Screening algorithm for inclusion of participants in the study

Of the 670 patients on routine care, 536 were screened for the HBsAg. Of these, 149 (27.8%) were seropositive for the hepatitis B surface antigen (HBsAg+) and 387 (72.2%) were seronegative for the hepatitis B surface antigen (HBsAg-). (Fig. 2).

### Demographic characteristics

A total of 387 apparently health HBsAg seronegative participants were recruited in the study. Majority of the participants were female (62.0%), aged ≥ 30 years (53.5%) and married (62.5%). A small number had history of blood transfusion (10.6%), alcohol use (18.1%), history of familial contact with an HBV infected person (10.6%), medical person (9%) with none with history of organ transplant. Other characteristics are shown in Table 1.

**Table 1:** General characteristics of the study participants

Characteristic	Outcome	Number	Percentage
Sex	Female	240	62.0%
	Male	147	38.0%
Age	> 30 years	207	53.5%
	≤ 30 years	180	46.5%
Marital Status	Married	242	62.5%
	Unmarried	145	37.5%
History of Blood Transfusion	No	346	89.4%
	Yes	41	10.6%
Alcohol use	No	317	81.9%
	Yes	70	18.1%
Contact with HBV infected person	No	338	87.3%
	Yes	49	12.7%
History of Organ transplant	No	387	100.0%
Injection Drug Use	No	338	87.3%
	Yes	49	12.7%
Born from a hospital facility	No	198	51.2%
	Yes	189	48.8%
History of infection with STD	No	276	71.3%
	Yes	111	28.7%
Medical person	No	352	91.0%
	Yes	35	9.0%
Total		387	100.0%

Abbreviations: HBV: - hepatitis B virus, STD: - sexually transmitted disease

### Serological Markers, Liver biochemistry tests and HBV viral load

None of the samples was positive for the HBeAg. For all the remaining markers, the seropositivity was significantly higher among the OBI participants compared to the HBsAg negative/HBV DNA negative participants (*p* < 0.05) (Supplemental file S1, Table 2). Serum bilirubin and the ALT were normal among the HBsAg/HBV DNA negative participants and slightly elevated among the OBI. The other parameters were only slightly elevated among both study groups. However, the levels of the parameters did not differ significantly by study group (*p* > 0.05) (Table 2). The viral load was detected in 21 samples with a mean ± SD of 194.4 ± 122.05 IU/mL and ranged from 71 to 542 IU/mL to give a 5.43% prevalence of OBI (Supplementary file S2, Table 2).

**Table 2:** Comparison of demographic and laboratory features of patients with OBI and participants negative for HBsAg and HBV DNA

Characteristic	OBI	HBsAg Negative and HBV DNA Negative	*P* value
Age ≤ 30 years, n (%)	13(61.9)	167(45.6)	0.2579
Male, n (%)	12(57.1)	135(37.2)	0.1767
AST; Mean ± SD (IU/L)	44.5 ± 38.5	39.90 ± 28.40	0.9018
ALT; Mean ± SD(IU/L)	46.6 ± 33.8	41.8 ± 59.7	0.2366
GGT; Mean ± SD(IU/L)	46.5 ± 37.7	48.7 ± 51.5	0.7098
Serum bilirubin; Mean ± SD (µmol/L)	16.4 ± 21.4	16.7 ± 16.5	0.1589
Anti-HBs Positivity, n (%)	13(61.9)	115(31.4)	0.0289*
Anti-HBe Positivity, n (%)	18(85.7)	53(14.5)	< 0.0001*
Anti-HBc Positivity, n (%)	21(100)	30(8.2)	< 0.0001*
Viral load (IU/mL),	194.4 ± 122.05		
Total, n (%)	21(5.43)	366(94.6)	

*p value < 0.05 statistically significant at 95%CI

## Discussion

Available information has implicated the OBI as a predictor of chronic hepatitis B virus (CHBV) infection, virus reactivation among the immune compromised and progression to hepatocellular carcinoma [3, 19, 20]. In this study, we report a prevalence of 21/387 (5.43%) which is comparable to the prevalence of 5.41% among the south African HIV patients [21] but lower than the pooled prevalence of 14.8% reported in a recent systematic review and meta-analysis in Africa [8], 17% in Nigeria [22], 15.1% in Sudan [23], 6.6% in Botswana [13], 30% previously reported by in Uganda [6], 8% global prevalence [7] and 5.56% in Ethiopia [24]. However, it is higher than the 1% prevalence reported in Cameroon among blood donors [25], 0.84% among blood donors in Egypt [26], 1.8% among the hemodialysis patients in Egypt [27] and 2.92% among chronic hepatitis C virus Egyptian patients [28]. The stringent inclusion criteria used by the different studies [13, 22, 23] and the OBI detection methods [24, 29] could explain the differences in the burden of OBI reported in the aforementioned studies and the current study. Besides, intermittent viremia has been reported [30] and the HBV DNA could be missed at the time of sampling. These challenges underscore the need for highly sensitive detection of HBV DNA leveraging on the recent advances in microfabrication and nanotechnology for use at the point of care [31] especially among the blood donors, pregnant woman on antenatal care and the immune compromised. Majority of the OBI cases (*n* = 11, 52.4%) had the viral load > 200IU/mL contrary to the cut off expected for the occult HBV DNA of 200IU/mL [2, 32]. This could be attributable to virus escape mutations leading to altered antigenic determinants during the HBsAg assay [33, 35].

We had more male with the OBI consistent with previous reports which have shown that the risk of the OBI is higher among men than the women [17, 34, 35]. Furthermore, we had more participants aged 18–30 years with the OBI compared to those aged 30 years and above. The findings in literature on age and the OBI risk have been inconsistent. For example, Said et al. [35], reported that age below thirty was a significant risk factor for prediction of the OBI among Egyetian blood donors. In contrast, Fopa et al. [36], reported an increase in the prevalence of the OBI with age. However, Minuk et al. [37], found out that age was not an identifier of the OBI in a community based study. The differences in the findings can be accounted for by the differenecs in design. For example, our study used hospital attendees, the study by Minuk et al. was community based while Said et al., and Fopa et al., used blood donors.

Finally, a relationship between the OBI and the prevalence of the HBcAb and HBsAb serological markers has been reported in this study in conformity with the findings by [38] and [39]. The prevalence of anti-HBc among the OBI patients has been accounted for by the OBI being a late phase of overt chronic hepatitis B virus infection [40]. The HBc anti-bodies (HBcAb), un like the HBsAb are non-protective during chronic infcetion and the HBcAb+/ HBsAg- is a marker of serological occult hepatitis B virus infection [41]. The prevalence of HBcAb reported in this study is lower than the prevalence of 15% [42] in Greece and 13.5% in Korea [43]. The low levels of HBcAb is predictive of infection with viral variants [44]. The prevalence of the ant-HBs among the OBI has been accounted for by the poor neutralising nature of the HBsAb following loss of recognition allowing for escape of the mutant virus from netralization even though the antibody may be present in neutralizing levels [45].

## Limitations

The HBV DNA levels > 200IU/mL could be attributable to immune escape mutations rather than OBI. To confirm this, sequencing is done. However, we were unable to do sequencing to establish these mutations which is the gold standard for the detection of the HBV genome from liver extracts. The presence of the HBV DNA in serum for the OBI occurs in flares and the viral load could have been underestimated. The cross-sectional nature of the study could have missed out some cases of the OBI leading to under estimation of the prevalence of OBI in our study.

## Conclusion and recommendation

In conclusion, silent HBV infections exist in this population risking transmission of HBV from mothers to the new born, reactivation among the immune compromised accelerating the development of the HCC, risking transfusion of infected blood and could explain the high endemicity of HBV. Therefore, surveillance for the presence of the OBI in the riskier groups should be routine by using the molecular diagnostics at the point of care (POC) leveraging from our experience of molecular TB diagnosis using the gene expert machine. Future research should focus on establishing burden of the OBI in riskier groups like the pregnant mothers on antenatal care and HIV-HBV co-infected in order to mitigate its adverse effects in these groups.

## Electronic supplementary material

Below is the link to the electronic supplementary material.

Supplementary Material 1

## Data Availability

All data generated or analyzed during this study are included in this published article and in the supplementally file.

## Abbreviations

ALT: Alanine aminotransferase
AOR: Adjusted odds ratio
AST: Aspartate aminotransferase
cccDNA: Covalently closed circular DNA
GGT: γ-glutamyl transferase
HBcAb: Hepatitis b core antibody
HBcAg: Hepatitis B core ant body
HBeAb: Hepatitis b precure antibody
HBeAg: Hepatitis B precure antigen
HBsAb: Hepatitis B surface antibody
HBsAg: Hepatitis B surface antigen
HBV: Hepatitis B virus
HCC: Hepatocellular carcinoma
NATs: Nucleic acid Amplification Techniques
NCBI: National Center for Biotechnology Information
OBI: Occult hepatitis B virus infection
WHO: World Health Organization
