# Supplementary material for: The status of occult HBV infection in a high endemic region: risk of community HBV transmission and reactivation
Source: BMC Res Notes. 2025 Jul 1;18:255. doi: 10.1186/s13104-025-07337-6 (PMC12211930; doi:10.1186/s13104-025-07337-6)
Supplement: Supplementary file 1 — Supplementary Material 1 [file 13104_2025_7337_MOESM1_ESM.pdf]

This questionnaire is intended to collect information of the risk factors that increase the susceptibility of people in a low and high endemic area

Patient information

	Sex		Age
Identifier No:	Male	Female	

Marital status	Education level
Single☐ Married☐ Widowed☐ Divorced ☐	Unknown ☐ Primary☐ Secondary☐ Post secondary☐

Risk factors leading to infection

1. Did you receive blood transfusion prior to 1989? Yes ☐ No ☐
2. Do you take alcohol? Yes ☐ No ☐
3. Did you receive an organ transplant prior to 1989? Yes ☐ No ☐
4. Have ever injected drugs not prescribed by the Doctor? Yes ☐ No ☐
5. Was the patient born from the health facility? Yes ☐ No ☐
6. Was the patient ever treated for a sexually transmitted disease? Yes☐ No ☐
7. Are you working in a medical field and you have blood exposure? Yes ☐ No ☐
8. Have you ever had a contact to a person who had hepatitis B? Yes ☐ No ☐
9. If yes, what was the contact type? Sexual ☐ Household ☐
   Others.......................... ....

Thank you for your cooperation and participation in the struggle for the fight against hepatitis B virus in Uganda

MAKERERE UNIVERSITY
SCHOOL OF BIOMEDICAL SCIENCES
APPROVED
VALID UNTIL .
★ 11 SEP 2020 ★
RESEARCH & ETHICS COMMETTEE
P. O. BOX 7072, KAMPALA
